# Comparison of the Efficacy of Male Sexual Activity Versus Alpha-Blockers in the Expulsion of Distal Ureteric Stones: A Systematic Review and Meta-Analysis

**DOI:** 10.7759/cureus.19347

**Published:** 2021-11-08

**Authors:** Charlotte Juman, Angus Bruce, Tsun Y Kwan, Anil Krishan, Syed Ali Mohsin Ehsanullah, Shehab Khashaba, Mohamed A Rafie

**Affiliations:** 1 Surgery, Walsall Healthcare NHS Trust, Walsall, GBR; 2 Urology, Walsall Healthcare NHS Trust, Walsall, GBR; 3 Surgery, University Hospitals Birmingham, Birmingham, GBR; 4 Urology, Shrewsbury and Telford Hospital NHS Trust, Shrewsbury, GBR; 5 Urology, University Hospitals Birmingham, Birmingham, GBR; 6 Urology, King Hamad University Hospital, Al Sayh, BHR

**Keywords:** masturbation, ejaculation, sexual intercourse, alpha-blocker, tamsulosin, urolithiasis

## Abstract

Globally, the prevalence of urolithiasis is increasing, with limited effective treatment options. Though debate exists within the literature, the use of medical expulsive therapy (MET) for distal ureteric stones in the form of alpha-blockers is commonplace. Alpha-blockers work via the inhibition of norepinephrine, resulting in a small degree of distal ureteric relaxation. Nitric oxide (NO), the main neurotransmitter involved in penile erection, causes smooth muscle relaxation of the distal ureter. It is hypothesised that these alternative pathways may achieve the same desire clinical effect.

To our knowledge, this is the first meta-analysis comparing the efficacy of male sexual activity, in the form of intercourse or masturbation, to alpha-blockers in the expulsion of ureteric stones.

We conducted a comprehensive search of electronic databases (PubMed, MEDLINE, EMBASE, SCOPUS, CENTRAL and Google Scholar), identifying studies comparing male sexual activity versus alpha-blockers, in male patients with distal ureteric stones. The Cochrane risk-of-bias tool was used to assess the included studies. For data analysis, a random effects model was used in the event of significant heterogeneity (>75%), with fixed-effects modelling in the event of low-moderate heterogeneity.

A search of electronic databases found three randomised control trials (RCTs), enrolling a total of 262 patients. There was no statistically significant difference observed when patients engaged in sexual activity rather than alpha-blocker, when looking at stone expulsion rate at two weeks (P=0.36), expulsion rate at four weeks (P=0.57), or the mean stone expulsion time (P=0.21). Furthermore, there was no significant difference observed when looking at analgesic requirements (P=0.43), or the requirement for additional procedures (P=0.57).

Our meta-analysis demonstrated that male sexual activity as an alternative therapy for distal ureteric stones had comparable outcomes to the use of alpha-blocker, proving a viable alternative therapy in those patients wishing to avoid pharmacological management.

## Introduction and background

Globally, the prevalence of urolithiasis is increasing [1], with limited effective treatment options. About 12% of the world population will be affected at some stage in their lifetime [2]. Ureteric stones comprise 20% of all urinary tract stones, with 70% located in the distal ureter [3]. The increasing burden and impact of kidney stone disease on healthcare systems is considerable, with the resultant cost in England estimated to be between £190 million and £340 million in the year 2010 [4]. Furthermore, the impact on patient quality of life is substantial, with a recent world literature review finding seven of nine studies demonstrated a lower quality in life in those patients with kidney stone disease [5]. Guidelines surrounding the use of alpha-blockers in patients with distal ureteric stone differ throughout the world. The American Urological Association (AUA) advocates the use of alpha-blockers in patients with distal ureteric stones less than 10mm, while the European Association of Urology (EUA) suggests offering alpha-blockers as a treatment option for distal ureteric stones greater than 5mm [6,7]. The current National Institute for Health and Care Excellence (NICE) guidelines recommend the use of alpha-blockers for medical expulsive therapy of distal ureteric stones less than 10mm [8]. This guidance has become controversial and widely debated in recent years, following publication of the SUSPEND trial, the largest randomized controlled trial looking at the effect of tamsulosin as part of medical expulsive therapy. The trial concluded that there was no benefit in using alpha-blockers for ureteric stones [9]. In contrast, a more recent meta-analysis advocates the use of alpha-blockers in the management of ureteric stones [10]. With alpha-blockers under the microscope, there is a requirement for an alternative with a substantial evidence base to support it.

Nitric oxide (NO), the main neurotransmitter involved in penile erection, causes smooth muscle relaxation of the distal ureter [11-13]. Hence, several recent studies have looked into the role of sexual intercourse or masturbation versus placebo in the treatment of ureteric stones. A 2019 meta-analysis comparing three such randomised controlled trials (RCTs) concluded that, compared with placebo, sexual intercourse exhibited greater efficacy for the treatment of distal ureteral stones, whilst potentially alleviating pain [3]. However, this meta-analysis did not reflect the real-life practice of alpha-blockers as part of the medical expulsive therapy for stones.

To our knowledge, this is the first meta-analysis comparing the efficacy of male sexual activity, in the form of intercourse or masturbation, to alpha-blockers in the expulsion of ureteric stones. We found it pertinent to perform a systematic review and meta-analysis of all available studies to evaluate male sexual activity as an alternative to alpha-blockers in the management of distal ureteric stones.

## Review

Methods

The protocol of this review was decided prior to the search strategy and registered in the International Prospective Register of Systematic Reviews (PROSPERO) with the registration number CRD42021232091.

The study was performed and presented according to Preferred Reporting Items for Systematic Reviews and Meta-Analyses (PRISMA) standards [14].

Eligibility Criteria

All RCTs and comparative observational studies evaluating the outcomes of male sexual activity (in the form of either sexual intercourse or masturbation) versus alpha-blockers in the treatment of distal ureteric stones were considered eligible, irrespective of original language. The study population of interest consisted of adult men of any age with distal ureteric stones of any size. Any studies which included female patients, mid-ureteric stones or proximal ureteric stones were excluded. Male sexual activity was considered as the intervention of interest and any alpha-blocker was considered as the comparator.

Outcome Measures

The efficacy of each treatment was considered as the primary outcome measure. Efficacy was measured by stone expulsion at two weeks, stone expulsion at four weeks and expulsion time. The secondary outcome measures included analgesic requirement and need for additional intervention.

Systematic Search Strategy

In accordance with our pre-defined standards for study selection, a comprehensive search strategy was performed by two independent authors through PubMed, MEDLINE, EMBASE, SCOPUS, CENTRAL and Google Scholar. The last search was run on the 19th of January 2021. Table 1 displays the literature search strategy.

**Table 1:** Literature search strategy ti,ab,kw = terms in either title or abstract or keyword fields

Search No	Search strategy^*^
#1	MeSH descriptor: [sexual intercourse] explode all trees
#2	Sexual intercourse : TI,AB,KW
#3	MeSH descriptor: [masturbation] explode all trees
#4	Masturbation: TI,AB,KW
#5	#1 OR #2 OR #3 OR #4
#6	MeSH descriptor: [ureteric stone] explode all trees
#7	Ureteric stone: TI,AB,KW
#8	MeSH descriptor: [urolithiasis] explode all trees
#9	Urolithiasis: TI,AB,KW
#10	#6 OR #7 OR #8 OR #9
#11	MeSH descriptor: [tamsulosin] explode all trees
#12	Tamsulosin: TI,AB,KW
#13	MeSH descriptor: [alpha blocker] explode all trees
#14	Alpha blocker: TI,AB,KW
#15	#11 OR #12 OR #13 OR #14
#16	#5 AND #10 AND #15
* This search strategy was adopted for following databases: MEDLINE, EMBASE, CINAHL and the Cochrane Central Register of Controlled Trials (CENTRAL)	

To identify suitable studies, the title and abstract were assessed initially by two independent authors. The full text of relevant articles were obtained and evaluated against the aforementioned criteria of inclusion. If discrepancy arose between the two searching authors, the opinion of a third author was sought to reach a majority verdict.

Screening of the references of the included articles was assessed for any further articles to be included in the analysis.

Data Extraction

In accordance with Cochrane recommendations for intervention reviews, an electronic sheet was made in order to extract data. Data extraction was performed by two independent authors. Data collected included study-related data (first author, year of publication, country of origin, journal, study population, comparison of interest), baseline patient demographics of the study population (age, stone size), and outcome data.

Summary Measures and Synthesis

Review Manager 5.3 software was used for data synthesis, with random effects modelling employed for data analysis. All results are displayed using a forest plot with 95% confidence interval (CI).

For each continuous variable, the mean difference (MD) between each group was calculated. For each dichotomous variable, the odds ratio (OR) was calculated.

The between-study heterogeneity was assessed and calculated using the I2 statistic. 0 to 50% represented mild heterogeneity, 50 to 75% moderate heterogeneity, while 75 to 100% a significant or high level of heterogeneity.

For each included dichotomous variable, the pooled OR, risk ratio (RR) and risk difference (RD) was calculated to determine any variation in the direction of pooled effect size. For dichotomous differences, the MD and standard mean difference (SMD) were also calculated, to observe any potential change in pooled effect size. Leave-one-out sensitivity analysis was performed, to assess the influence of each study. This was done by removing one study at a time and observing the resultant effect. For data analysis, a random effects model was used in the event of significant heterogeneity (>75%), with fixed-effects modelling in the event of low-moderate heterogeneity.

Results

**Figure 1:**
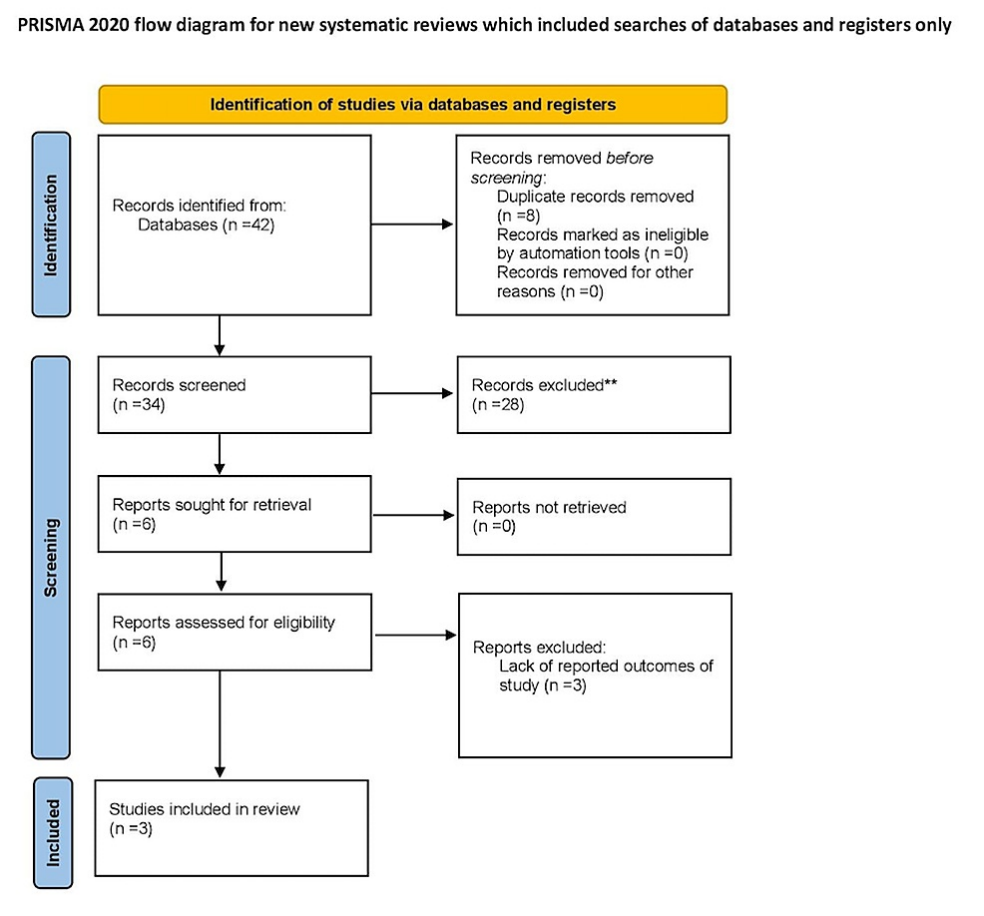
Preferred Reporting Items for Systematic Reviews and Meta-Analyses (PRISMA) Flow Diagram

Our comprehensive search strategy identified 34 articles. Thirty-one were removed for not fulfilling the inclusion criteria, or the absence of outcomes defined for the purpose of the study. Ultimately, three studies met the eligibility criteria (Figure 1) [15-17]. These included a total of 262 patients, with 140 in the sexual activity group, and 122 in the alpha-blocker group. Table 2 contains a summary of the study populations, while Table 3 contains the demographic information of the included studies.

**Table 2:** Study-related data RCT: randomised controlled trial

Author	Year	Country	Journal	Type of study	Study population	Comparison
Bayraktar [15]	2017	Turkey	Int Urol Nephrol	RCT	Male; distal ureteric stone (5-10mm)	Sexual intercourse (at least 3-4x/week) vs Tamsulosin (400mcg/day)
Dolouglu [16]	2015	Turkey	Urology	RCT	Male; distal ureteric stone (≤6mm)	Sexual intercourse (at least 3-4x/week) vs Tamsulosin (400mcg/day)
Turgut [17]	2020	Turkey	Int Urol Nephrol	RCT	Male; distal ureteric stone (5-10mm)	Masturbation (at least 3-4x/week) vs Tamsulosin (400mcg/day)

**Table 3:** Baseline demographic data

Author	No of patients	Ejaculation	Alpha-blocker	Mean age (years)	Stone size (mm)
Bayraktar [15]	126	66	60	38.66 vs 34.4 (p=0.909)	7.01 vs 7.09 (p=0.7492)
Dolouglu [16]	52	31	21	34.9 vs 39.3 (p=0.07)	4.7 vs 5 (p=0.4)
Turgut [17]	84	43	41	37 vs 37.6 (p=0.779)	6.93 vs 7.1 (p=0.406)

Methodologic Quality and Risk of Bias

**Figure 2:**
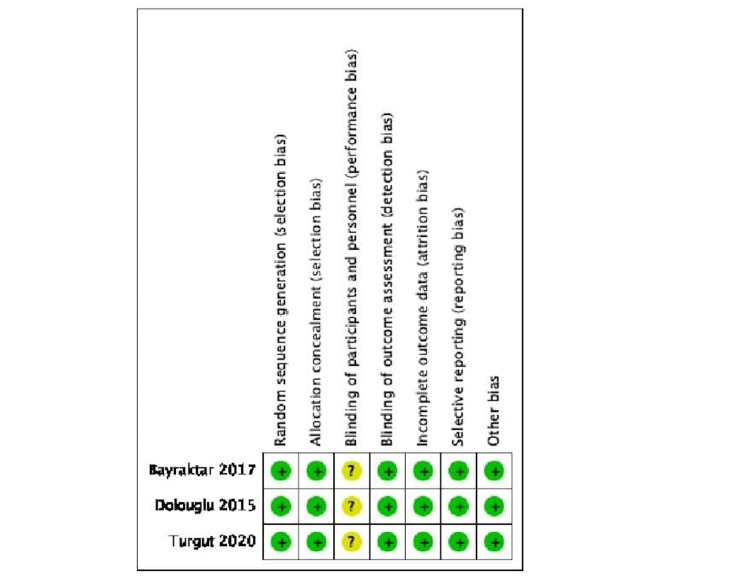
Methodological quality assessment

Two independent authors each evaluated the methodological quality and risk of bias of each of the included studies, using the Cochrane tool for RCTs. The results of the methodological quality assessment are displayed in Figure 2. The quality of evidence was high, with low risk of bias across each domain (selection bias, attrition bias, reporting bias, and other bias), aside from performance bias. This is due to the inability to achieve the blinding of the participants in this type of RCT.

Primary Outcome (Efficacy)

Efficacy of each treatment was assessed by measuring the stone expulsion at two weeks post-presentation, four weeks post-presentation and the mean expulsion time.

**Figure 3:**
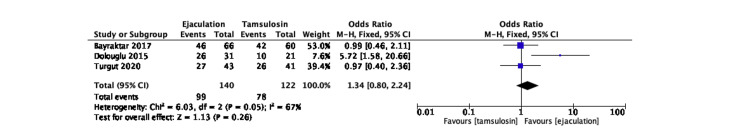
Forest plot of stone expulsion at 2 weeks

The stone expulsion rate at two weeks in the sexual activity and alpha-blocker groups was 70.7% and 63.9% respectively. There was no statistically significant difference in stone expulsion at two weeks between the two groups (OR 1.34, 95% CI 0.80-2.24, P=0.26) (Figure 3). A moderate level of heterogeneity existed among the included studies (I2=67%, P=0.05).

**Figure 4:**
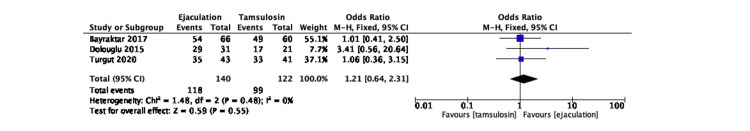
Forest plot of stone expulsion at 4 weeks

The stone expulsion rate at four weeks in the sexual activity group was 84.3%, and 81.1% in the alpha-blocker group. There was no statistically significant difference in stone expulsion between the two groups (OR 1.21, 95% CI 0.64-2.31, P=0.55) (Figure 4). A low level of heterogeneity existed among the included studies (I2=0%, P=0.48).

**Figure 5:**
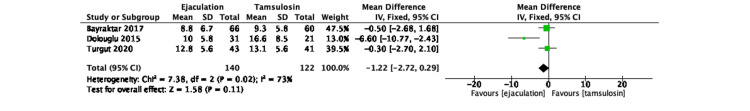
Forest plot of mean expulsion time

The mean expulsion time in the sexual activity group was 10.3 days in the ejaculation group and 11.8 days in the alpha-blocker group. There was no statistically significant difference in the expulsion time between the two groups (MD -1.22, 95% CI -2.72-0.29, P=0.11) (Figure 5). A moderate level of heterogeneity existed among the included studies (I2=73%, P=0.02).

Secondary Outcomes

The secondary outcomes measured were analgesic requirements and the requirement for further operative intervention.

**Figure 6:**
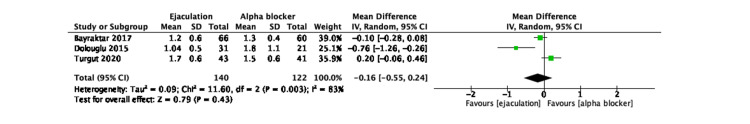
Forest plot of analgesic requirements

The mean use of as-required diclofenac was 1.32 injections per day in the sexual activity group and 1.45 injections per day in the alpha-blocker group. There was no statistically significant difference in the analgesic requirements between the two groups (MD -0.16, 95% CI -0.55-0.24, P=0.43) (Figure 6). A high level of heterogeneity existed among the included studies (I2=83%, P=0.003).

**Figure 7:**
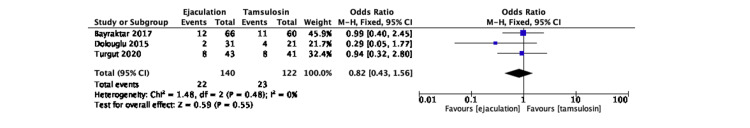
Forest plot of requirement for additional intervention

The requirement for subsequent additional intervention was 15.7% in the sexual activity group, and 18.9% in the alpha-blocker group. There was no statistically significant difference in the requirement for additional intervention between the two groups (OR 0.82, 95% CI 0.43-1.56, P=0.55) (Figure 7). A low level of heterogeneity existed among the included studies (I2=0%, P=0.48).

Sensitivity Analysis

The use of switch from fixed or random-effects modelling, as opposed to the chosen one in each case based on heterogeneity, did not significantly alter any calculated outcomes. Similarly, the direction of pooled effect remained unchanged when OR, RR or RD were calculated from dichotomous variables, nor did it differ when MD or SMD were calculated from continuous variables. Leave-one-out sensitivity analysis did not alter any reported outcomes.

Discussion

Our systematic review and meta-analysis assessed the efficacy of male sexual activity, in the form of sexual intercourse or masturbation, as compared to alpha-blockers in the treatment of distal ureteric stones. Three comparative RCTs, enrolling a total of 262 patients, were included. Treatment efficacy was defined by stone expulsion at two and four weeks, and stone expulsion rate, with secondary outcomes of analgesic requirement and need for additional intervention. Across all of these outcomes, male sexual activity was found to be comparable to alpha-blockers, with no statistically significant differences found between treatment groups. Interestingly, male sexual activity was favoured in all of these outcomes; although there was no statistical significance.

The efficacy of alpha-blockers as medical expulsive therapy (MET) in the treatment of ureteric stones has been thrown into question in recent years. The SUSPEND trial, which sought to establish the benefit of alpha-blockers (tamsulosin) and calcium channel blockers (nifedipine) in the management of ureteric stones, showed no significant impact on the requirement for intervention at four weeks, nor pain limitation [9]. The subset analysis from the trial showed a trend towards benefit of tamsulosin for lower ureteric stone >5mm in size. Despite this, the ongoing use of alpha-blockers for distal ureteric stones continues to be advocated by the EAU, AUA and NICE [6-8]. The conflict in evidence raises concerns about the off-label use by urologists of alpha-blockers as part of MET.

Tamsulosin can prove an undesirable choice for men who want to avoid pharmacological management and resultant adverse effects. Retrograde ejaculation was a resultant side effect experienced by 8-18% of users, while other frequently occurring side effects include orthostatic hypotension, dizziness, headache and diarrhoea [18,19]. Floppy iris syndrome can complicate cataract surgery in patients who have previously used tamsulosin and last for a lifetime, even after a single dose [20].

The distal ureter contains alpha-1 adrenergic receptors, the blockage of which has been proven to decrease frequency of peristalsis and the basal ureteral tone [16]. It is on this basis that alpha-blockers have emerged as the first-line for medical expulsive therapy. The presence of nitrergic fibres has also been established in the ureter, in both human and porcine specimens [21,22]. Released and administered NO has been shown to relax porcine intravesical ureter [22,23]. NO is the main neurotransmitter involved in penile erection and sexual intercourse. Doluoglu et al. [16] hypothesise that the distal ureter could be stimulated by nitrergic nerve endings during sexual intercourse, resulting in higher stone expulsion rate.

In keeping with this hypothesis, Xu et al. [3] conducted a meta-analysis comparing sexual intercourse with placebo in the management of distal ureteric stones. Their results showed that sexual intercourse three or four times a week increased the expulsion rate of distal ureteric stones at two and four weeks and decreased analgesic requirements compared with placebo. These findings, in addition to those found in our own study, hold strength in the argument for sexual activity to become a genuine alternative therapy in male patients with distal ureteric stones. This will avoid any adverse effects of alpha-blocker therapy.

Our review and reported outcomes have limitations. Firstly, the study size of each included study was relatively low, resulting in a total of 262 patients included, raising the possibility of a type 2 statistical error. A lack of high-quality comparative studies published comparing sexual activity to alpha-blockers resulted in the inclusion of only three studies (though all RCTs), with each study performed within five years of the others. Furthermore, our study included only the male population. Of note, a single study evaluated the efficacy of sexual intercourse in the expulsion of distal ureteric stones in an entirely female population [24]. When compared to symptomatic treatment only, sexual intercourse was found to increase the spontaneous passage of stones, whilst also reducing analgesic requirements. Further studies are required to further look into separate cohorts of patients with different stone size and locations. In future studies, it would be recommended to include patient stone passage history, due to the large impact it will likely play on the future stone passage abilities.

## Conclusions

Our meta-analysis demonstrated that male sexual activity as a form of alternative therapy for distal ureteric stones had comparable outcomes to the use of alpha-blockers. We found no statistically significant difference in stone expulsion rates, analgesic requirements or the future need for additional therapy. The evidence base for alpha-blockers as a pharmacological treatment for distal ureteric stones is well-established, though the side-effect profile can make the choice undesirable. Though further research is required, our results show promise for male sexual activity to emerge as a viable alternative therapy, in those patients wishing to avoid pharmacological management.
